# Multidisciplinary Application of an External Tissue Expander Device to Improve Patient Outcomes: A Critical Review

**DOI:** 10.1089/wound.2019.1112

**Published:** 2020-09-17

**Authors:** Brendan J. MacKay, Anthony N. Dardano, Andrew M. Klapper, Selene G. Parekh, Mohsin Q. Soliman, Ian L. Valerio

**Affiliations:** ^1^ Department of Orthopaedic Surgery, Texas Tech University Health Sciences Center, Lubbock, Texas.; ^2^ Department of Orthopaedic Surgery, University Medical Center, Lubbock, Texas.; ^3^ Department of Plastic and Reconstructive Surgery, Florida Atlantic University, Charles E. Schmidt College of Medicine, Boca Raton, Florida.; ^4^ Department of Orthopaedic Surgery, North Carolina Orthopaedic Clinic, Durham, North Carolina.; ^5^ Fuqua Business School, Duke University, Durham, North Carolina.; ^6^ Overland Park General and Bariatric Surgery, HCA Physician Services, Overland Park, Kansas.; ^7^ Department of Plastic and Reconstructive Surgery, Ohio State University Wexner Medical Center, Columbus, Ohio.

**Keywords:** continuous external tissue expansion, tissue expansion, complex wound, traumatic wound, fasciotomy, delayed primary closure

## Abstract

**Significance:** Continuous external tissue expansion (CETE) is a versatile tool in soft tissue injury management, and could be an addition to the traditional reconstructive ladder.

**Recent Advances:** This critical review discusses the principles and application of CETE, covering a company-sponsored consensus meeting on this emerging technology and highlighting the DermaClose^®^ (Synovis Micro Companies Alliance, Inc., Birmingham, AL) device's unique approach to soft tissue injury management. There is clinical evidence to support the use of CETE in the management of a number of wound types, including fasciotomy, trauma, amputation, and flap donor sites. The device can be applied to open wounds, potentially avoiding the need for a skin graft or other more complex or invasive reconstruction options. DermaClose applies constant tension without restricting blood flow and does not require repeated tightening.

**Critical Issues:** CETE is becoming more widely used by surgeons of different specialties, and numerous reports describing its efficacy and safety in wound management have been published. Surgeons using CETE must follow the correct technique and select patients carefully to achieve optimal outcomes. However, there is no single source of information or consensus recommendations regarding CETE application.

**Future Directions:** Prospective evidence on the efficacy and safety of CETE in clinical practice is required to communicate the best techniques and share important experiences. This will help to solidify its place in the reconstructive ladder as a valuable additional option for surgeons.

**Figure:**
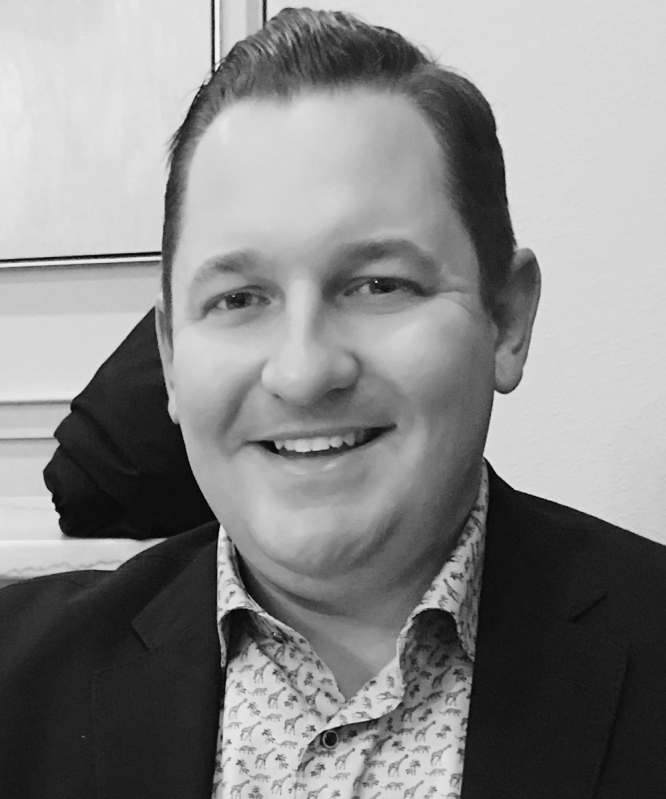
**Brendan J. MacKay, MD**

## Scope and Significance

This company-sponsored critical review discusses the role of a continuous external tissue expansion (CETE) device (DermaClose^®^; Synovis Micro Company Alliance Inc. [a subsidiary of Baxter International, Inc., Deerfield, IL]) in soft tissue injury management. Clinical evidence in various reconstruction settings and the authors' recommendations for optimal CETE application are presented.

## Translational Relevance

CETE capitalizes on the viscoelastic properties of the skin. It allows surgeons to achieve primary closure in a range of wound types that are not primarily closable, avoiding the need for more complex procedures.

## Clinical Relevance

Although CETE is becoming more widely used by surgeons from different specialties, information and recommendations regarding its use are limited. Using the correct technique results in fewer complications and improved outcomes for patients. Given the paucity of published data, this critical review may serve as a meta-analysis for CETE as it details methods for CETE application, clinical evidence for its use in a number of settings, and the authors' recommendations, specifically related to DermaClose, based on their clinical experience.

## Background

**Figure 1.**
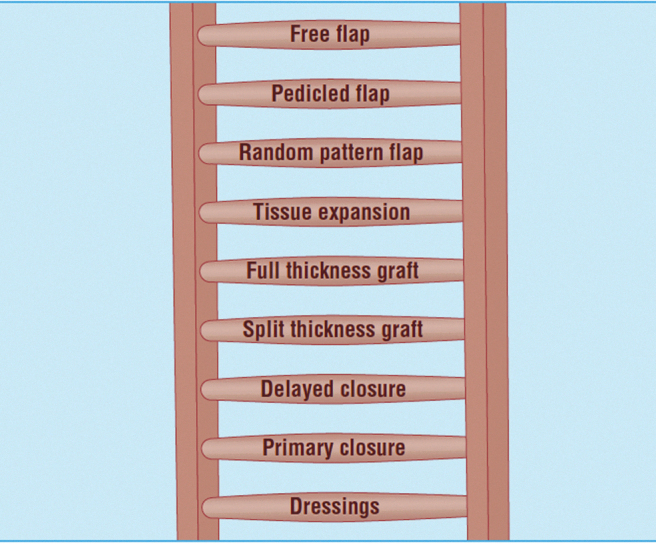
The reconstructive ladder.^1^ Traditional reconstructive techniques are arranged with increasing complexity higher up the ladder. CETE could be positioned below split thickness graft. Reproduced from ABC of wound healing: Reconstructive surgery,^1^ with permission from BMJ Publishing Group Ltd. CETE, continuous external tissue expansion.

The hierarchy of surgical techniques traditionally used by reconstructive surgeons is depicted in the “reconstructive ladder” (Fig. 1).^1^ Surgical developments, like CETE, may provide opportunities to add new rungs to the traditional reconstructive ladder. Furthermore, the simplicity of these devices makes them accessible to subspecialties outside plastic surgery.

CETE uses the viscoelastic properties of the skin. Mechanical strain induces cellular proliferation, leading to an increase in tissue surface area through generation of new tissue.^2^ Although the exact mechanism by which mechanical strain induces proliferation of new tissue is still being elucidated, several factors have been linked to stress and/or mechanical strain^2^ (Table 1).

**Table 1.** Mechanisms by which mechanical strain may affect cell proliferation in wound healing

Biochemical pathways
Multiple biochemical pathways are involved in the proliferation of new tissue. For example, chemical agents inhibiting cellular attachment to the ECM have been shown to cause tissue expansion, indicating that extracellular forces have a role in inducing cell growth. This discovery led researchers the discovery that mechanical strain affects signaling pathways in the proliferation cascade. Normal cell growth and cell proliferation are governed by many of the same growth factors including EGF, basic fibroblast growth factor, platelet-derived growth factor, and angiotensin II. Unsurprisingly, mechanical strain has been shown to stimulate increased expression of these growth factors and/or elevate sensitivity to their effects^3^
*ECM*
The ECM also plays an essential role in inducing cell proliferation needed to heal wounds. Mechanical strain has been shown to trigger signaling cascades by altering the ECM. Elevated collagen production increases phospholipase C activity, Ca^++^ mobilization, and inositol phosphate formation, all of which play an integral role in delivering signals that stimulate cell growth. Integrins found in the ECM cluster around growth-stimulating factors, resulting in cell mitosis. Morphologic changes in actin filaments modulate protein kinases, second messengers, and nuclear proteins. Actin filament bundles also interact with adjacent cells through cadherins, which may allow microfilaments to exchange signals under stress conditions^3^
*Ion channels*
Ion channels may also play an important role in wound healing. Influx of Ca++ ions stimulate K+ (feedback monitors) to prevent excess contraction and maintain membrane potentials. EGF is thought to be linked to the movement of K+ ions, and thus may be the mechanism by which ion channels affect cell proliferation^3^
*Protein kinases*
Protein kinases, particularly protein kinase C, are known to be involved in signal transduction pathways that stimulate tissue growth. These and other protein kinases such as phospholipase C and diacylglycerol are thought to interact with extracellular components to stimulate cell proliferation under stress conditions^3^
*Protein synthesis*
Typically, protein synthesis is inversely proportional to cAMP concentration, as cAMP is consumed in the process of making new proteins. When tissue is under stress, however, prostaglandin E_2_ has been shown to function as a substitute for cAMP^3^

cAMP, cyclic adenosine monophosphate; ECM, extracellular matrix; EGF, epidermal growth factor.

The majority of data on expanded skin is drawn from porcine models because of their similarity to human skin. One study evaluated expanded pig skin 10 days after removal of inflated silicone tissue expanders. Knight *et al.* reported a 47% increase in surface area, 9% reduction in thickness, and 9.3% increase in collagen content of the dermis. Tensile strain is thought to stimulate fibroblasts to increase collagen production.^3^

Two porcine studies evaluated the effects of an external negative pressure expander, and found that the area of expanded skin under negative pressure thickened. Histological staining suggested increased cell proliferation. Expanded tissue also showed increased blood vessel density, layered smooth muscle cells, and levels of growth factors involved in the angiogenic cascade.^4,5^

In 1984, Radovan described tissue expansion by subcutaneous implantation of silicone envelopes.^6^ This technique has been used in numerous settings with success rates up to 96.7%^6–9^ (Table 2).

**Table 2.** Advantages and disadvantages of various tissue expansion devices

Internal Tissue Expansion Device	Method	Advantages	Disadvantages
Silicone envelope	Silicone envelope is implanted, and saline is progressively injected into the implant to expand overlying tissue	Ability to match flaps to the recipient site. Avoids more complex distant and/or free tissue transfer flaps. Maintain localized sensation	Invasive implant. Relatively high complication rates
*External Tissue Expansion Device*	*Method*	*Advantages^a^*	*Disadvantages*
External Tissue Extender	1 cm wide polyamide straps inserted through the skin and attached to silicone holding bars. Strips have a stopper on one end and one-way locking device at the other	Simple, easy to apply and adjust	Frequent breakage and necrosis of skin bridges. Requires a crude estimation of the force skin can sustain without damage. Needs repeated manual tightening
SureClosure^®^	Stainless steel needles threaded through wound edges. Two U-shaped polycarbonate arms hook onto the needles on each side of the wound. U-shaped arms are secured on threaded polycarbonate bars running across the wound. Screw heads on one end the threaded bars are turned to tighten, and a ruler runs across the wound to measure progress	Does not undermine skin at wound edges. Simple and easy to apply	Requires a crude estimation of the force skin can sustain without damage. Needs repeated manual tightening
TopClosure^®^	Shape-adaptive attachment plates are glued to skin, with holes for optional invasive attachment. A ratchet strap runs through the attachment plates, one of which has a lock/release mechanism	Can be applied noninvasively. Useful in treating longstanding open wounds	Requires a crude estimation of the force skin can sustain without damage. Needs repeated manual tightening
DynaClose^®^	Multiple strips of elastomer with adhesive fabric tape on either side are applied across the wound. These apply continuous tension to tissue at wound edges	Acts dynamically, moving as skin is stretched, applying a constant, cyclic stretching force. Useful in treating longstanding open wounds	Strips must be regularly changed to maintain continuous traction until the wound is closed
ABRA^®^	Elastomer bands are inserted through the skin and secured with contoured anchors on either side of the wound. Elastomers are adjusted (using marks on the bands for reference) to 1.5 times their untensioned length	Bands are marked to indicate appropriate tension. Acts dynamically, moving as skin is stretched, applying a constant, cyclic stretching force. Useful in treating long-standing open wounds	Requires undermining of wound edges. While bands are marked, visual approximation is still used to determine appropriate tension. Needs repeated manual adjustment
DermaClose^®^	Anchors are placed 0.5–1 cm from wound edges and secured to skin with staples. A nonelastic tension line originating from the tension controller knob is looped through the anchors in an X-pattern (Fig. 3). The tension controller knob is then turned clockwise until the constant force spring engages the clutch mechanism, preventing overtightening. Constant force is maintained through the spool as the wound is closed	Simple and easy to apply. Maintains exact maximum tension that does not cause skin necrosis or damage. Constant tension is maintained without the need for repeated adjustment. Useful in treating longstanding open wounds	Requires undermining of wound edges

^a^ All external tissue expanders offer the advantages of the internal expansion device in addition to those listed.

External skin expanders can be used on problematic wounds not amenable to direct closure. Method of expansion, advantages, and disadvantages for different devices are given in Table 2. Some methods (rubber bands and skin staples; DynaClose^®^; TopClosure^®^) use bands or strips attached to the skin around the defect, exerting pressure to draw the wound edges together.^11–13^

In 1993, Blomqvist and Steenfos described a device using subcutaneous straps placed under a skin defect, connected with holding bars and a locking device.^14^ The historical SureClosure^®^ device included a tension indicator for precise pressure application.^15^ The ABRA^®^ Surgical device, comprising anchors connected across a wound with elastomers, adopts a similar method of manual tension setting.^16^ All these methods require repeated adjustment and/or replacement (Table 2).

**Figure 2.**
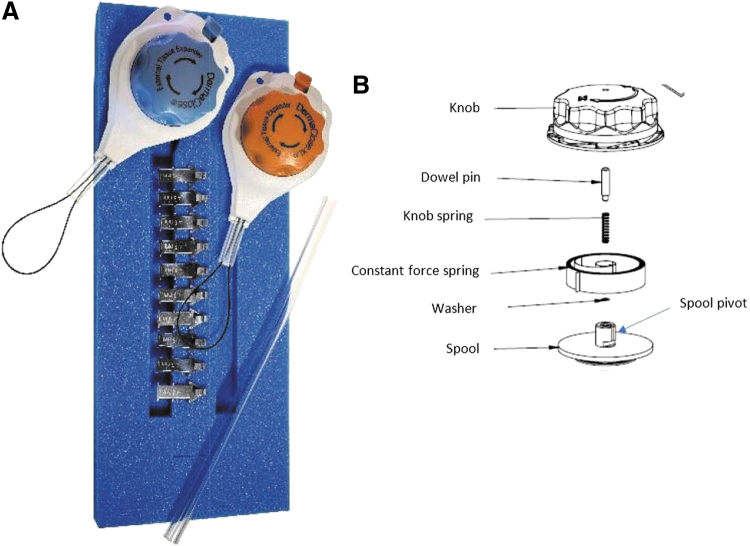
**(A)** Components of the CETE device, including skin anchoring pleats, a tension controller containing a tension line to be placed under a consistent strain or force, and bridge tubing. **(B)** When the DermaClose tension controller knob is turned clockwise, the constant force spring coils up until a clutch mechanism engages that prevents the pulling force from exceeding 11.7 N (1.2 kg). The constant force spring maintains its force to the spool of tension controller line as the wound edges are gradually approximated.

More recently, a CETE device that maintains tension at 11.7 N (1.2 kg), a tension that does not cause skin necrosis or damage, has been developed (Fig. 2).^17^ No manual tightening is required.^18–21^ The device can be applied to open wounds, potentially avoiding more complex or invasive reconstruction options.^22^

## Discussion

### Method

The authors were selected for their experience with CETE. A company-sponsored consensus group was formed, and a literature search was performed in PubMed to identify publications concerning tissue expansion and CETE.

### Correct technique for continuous external tissue expansion

**Figure 3.**
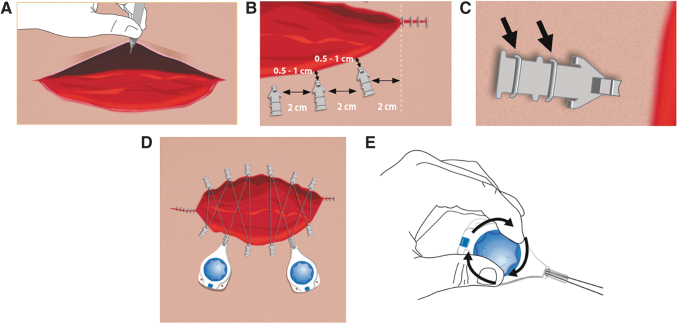
Correct method for application of CETE device.^10^ One controller and six skin anchors should be used per 10 cm wound length. Additional anchors and controllers may be used for larger wounds. **(A)** Before applying the device, undermine or elevate the skin wound margins and close as much of the wound as possible. **(B)** Place skin anchors 0.5–1 cm from the wound edge, and ∼2–3 cm apart. **(C)** Secure the skin anchors with two standard wide skin staples (6–7 mm). **(D)** Place a barrier dressing on the wound bed and under the wound margins. Xeroform^®^ (petrolatum impregnated gauze), DuoDERM, or other barrier dressings may be placed on the wound bed and under the wound margins before attaching the tension controller line to the skin anchors. Place the tension controller at the widest part of the wound and thread the tension line through the skin anchors in a shoelace pattern. The tension controller can be loosely secured to the skin with either sutures or tape. Placing a protective barrier (*e.g*., DuoDERM) under the controller is recommended to minimize chances of skin blistering, compression, and ulceration. Gauze should not be placed under the tension controller as this can cause blistering. The wound may be dressed as required. **(E)** Turn the blue dial on the controller clockwise until a click is heard, then lock the controller by pressing the button. When the tension is set and the controller locked, no additional tightening is required.

**Figure 4.**
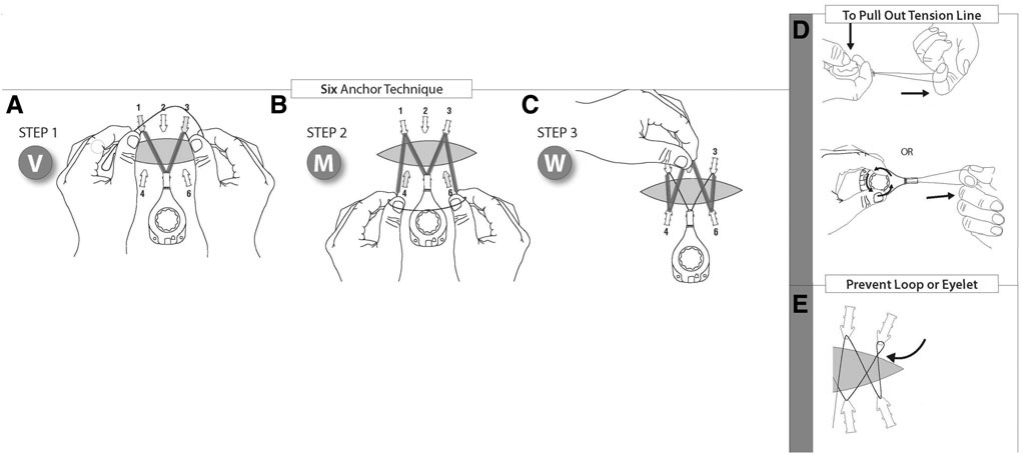
Six anchor lacing technique^23^: **(A)** Grasp the tension line with both hands near the front of the tension controller, and form the letter V by going around the tabs of anchors nos. 1 and 3. **(B)** With the next movement, form the letter M by looping the tension line around tabs nos. 4 and 6. **(C)** Guide the line over anchor tab no. 2 opposite the tension controller, holding the tension line with one hand to complete the letter W. Gently pull back on the tension controller to remove any slack in the line. Turn the blue control knob clockwise until clicking is heard (approximately 22 half rotations). **(D)** To pull out tension line, press down on the tension controller knob or turn the knob counter-clockwise while pressing down on the knob. **(E)** Take caution to prevent loops/eyelets from forming in the tension line.

**Figure 5.**
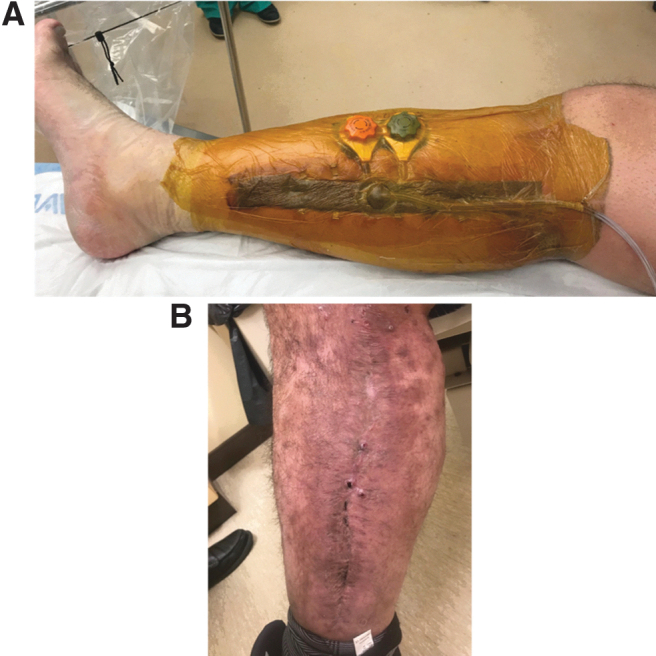
**(A)** The 46-year-old man with previous fasciotomy initially closed with split-thickness skin grafting 5 years postclosure, presented for options to address painful, tethered skin grafts. The skin graft was excised and the CETE device was placed. Image shows immediate postoperative appearance of CETE in combination with NPWT. **(B)** Wound after tissue expansion and delayed closure. Image courtesy of I.L.V. NPWT, negative-pressure wound therapy.

CETE is indicated for assisting with closure of moderate to large surgical or traumatic acute full-thickness wounds of the skin by approximating and reducing the size of the wound. Relative contraindications are ischemic, infected, or excessively fragile tissue.^17^ Caution should be exercised in patients who are immunocompromised, currently taking steroids, or have concomitant conditions affecting tissue quality. See Figs. 3–5 for the correct application method.^17^

Negative-pressure wound therapy (NPWT) can be used in conjunction with CETE (Fig. 5); NPWT foam should be cut 50% smaller than the wound, and nonadherent dressing (petroleum gauze or similar material) placed over skin anchors and tension line. The foam can be placed underneath or over the tension line, or both. It is important that the line can continue to tighten.

Some wounds may benefit from rest periods and changing anchor locations after a couple of days. If the CETE device is left on for a long period, the wound should be irrigated to maintain moisture.

### General surgery

#### Abdomen

##### Considerations

The causes, features, and optimal management of abdominal wounds are diverse, and as a result there is no gold standard for treatment.^24^ Tissue expansion can be useful in managing abdominal wounds, allowing for delayed primary closure and recruitment of skin with increased vascularity.^24^ Complications associated with certain tissue expansion techniques in abdominal wounds include infection, evisceration, and hematoma.^24^

##### Clinical evidence

One of the expert contributors used DermaClose to assist in closure of 64 open abdominal wounds. Complete closure was obtained within 6–8 days, and there were no complications secondary to the device.

There is one publicly available case report describing a 60-year-old woman with spontaneous bacterial peritonitis, after a history of metastatic abdominal carcinoid.^25^ The abdomen was opened secondary to exploratory laparotomy, resulting in a 14 × 6 cm wound. DermaClose was applied and the incision healed without incident after 8 days.

A retrospective case series described using the Canica ABRA dynamic wound closure system for abdominal wounds in 23 patients.^26^ Fourteen patients achieved delayed primary closure. Average duration of system application was 40 days.

##### Consensus recommendations

In abdominal wounds, place a couple of stitches from the skin through the fascia to secure the skin anchors to the CETE device, instead of skin staples, as this assists with fascia expansion. The NPWT should be placed with the foam under the tension line.

#### Fasciotomy

##### Considerations

Acute compartment syndrome places the skin and the underlying tissues under significant pressure. Once pressure is surgically released, tissue may need time to recover before tension is applied. Timing is crucial, as tight skin closure could lead to further ischemia and necrosis.^27^ Edema may also prevent primary closure. The resulting wounds may be closed by secondary intention, delayed primary closure, split-thickness skin graft, or tissue transfer/flap.^27^ Although skin grafting remains a mainstay in many practices, the associated secondary contraction can cause painful tethered scars, decreased range of motion of the underlying muscle, and sensitivity to pressure or touch (Fig. 5).

**Figure 6.**
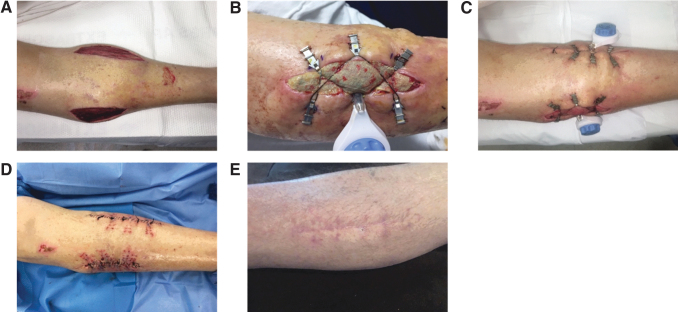
Postfasciotomy application of Dermaclose. **(A)** Wound postfasciotomy. **(B)** Day 3 postapplication of the CETE device, with Xeroform^®^ covering the exposed muscle. **(C)** Postoperative day 7, before closure. **(D)** Closure of both wounds. **(E)** Well-healed incision at 3-month follow-up. Photographs courtesy of A.N.D.

When indicated, techniques that facilitate delayed primary closure are preferred. Delayed primary closure is associated with better cosmetic appearance, less scarring, and improved motion of underlying muscles. It can achieve adequate soft tissue coverage over sensitive structures such as sensory nerves, and provides superior functional and clinical outcomes compared with skin grafting or more invasive techniques. Earlier closure may also save time and resources. This can be achieved through CETE in combination with NPWT, which helps reduce the underlying tissue edema.^27^ Clinicians have two options for CETE application: (1) Apply at the time of initial fasciotomy and leave untightened until skin has recovered. (2) Wash out the wound, place a vacuum dressing, and wait to place the device until a second look in the OR. Figure 6 shows postfasciotomy application of DermaClose.

##### Clinical evidence

Literature on the use of the CETE with fasciotomy wounds is limited. One case report describes a complicated fasciotomy after a gunshot wound to the left lower extremity.^28^ On postoperative day 7, primary skin closure of the lateral wound was performed and 2 DermaClose devices were placed on the medial wound. Final closure was successfully performed 8 days after DermaClose application, with minimal undermining and tension.

One review described favorable experience using CETE with concurrent vacuum-assisted closure (VAC) therapy to manage fasciotomy.^27^

##### Consensus recommendations

As mentioned previously, clinicians have two options for CETE application in delayed primary closure. One author's practice is to perform the fasciotomies, then apply a VAC dressing. As some cases may need further debridement, the cleats are usually attached only when the wound is ready for closure.

Use NPWT alone or in combination with CETE to facilitate edema and swelling reduction before closure. Nonadherent gauze can be used beneath the VAC foam.

CETE can be used before definitive closure to bring wound edges closer; it may then negate the need for a skin graft or reduce the size of graft needed.

#### Postsurgical wounds

##### Considerations

Postsurgical wounds vary enormously in size, complexity, and setting; each has specific considerations. Skin grafts may not be viable to achieve closure, or the resulting donor site wounds may be undesirable. In revision surgery, surgical site tissue may be of poor quality.

Delayed closure after surgery can help control tissue edema and avoid the need for secondary procedures.

##### Clinical evidence

A case report described application of CETE to manage sternal wound dehiscence.^29^ A 58-year-old woman with several comorbidities experienced postoperative sternal wound infection after median sternotomy for aortic valve replacement. After 4 days of wound VAC, skin and subcutaneous fat were undermined and DermaClose was applied for 7 days. A soft foam dressing was placed underneath the device to protect underlying skin, but some blistering on the breasts occurred; this healed fully with local wound care. The wound was completely healed by the 5-month follow-up. The report noted benefits of CETE over NPWT including shortened time to definitive closure, reduction in costs, fewer dressing changes, and shortened hospital stay.

Use of DermaClose after Mohs micrographic surgery for removal of malignant proliferating trichilemmal tumor has been described.^21^ The Mohs procedure resulted in a 6.3 × 5.6 cm, which was then reduced to 1.5–1.0 cm after application of the device.

##### Consensus recommendations

For different wounds, the CETE device may be placed in a different position, or used as an adjunct with VAC, rotational flaps, or dermal substitutes. Multiple devices may be placed in different vectors. The optimal technique depends on the final goal of wound management.

CETE may be considered in complex postsurgical wounds with potentially mobile skin that may be amenable to closure without flap coverage.

**Figure 7.**
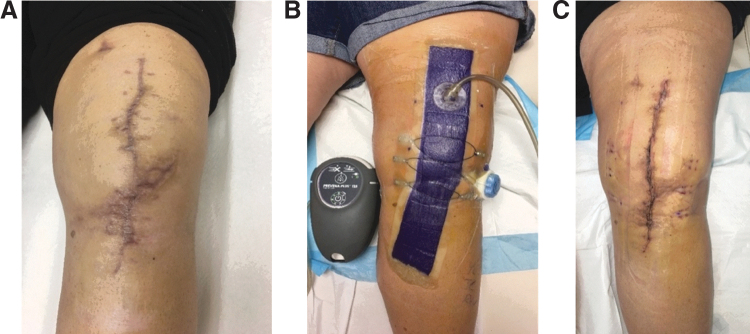
Use of the CETE device to off-load tension at time of primary closure. The patient had undergone several previous knee revisions with wound-healing difficulties. **(A)** Patient's knee prior to surgery involving application of CETE. **(B)** At this surgery, tension of the wound closure was offloaded with NPWT and CETE to avoid dehiscence and minimize wound complications. **(C)** Healed wound following CETE application. Photographs courtesy of A.N.D.

In revision surgery, CETE may be used to off-load tension on the primary repair to prevent dehiscence (Fig. 7).

### Foot and ankle

#### Total ankle arthroplasty

##### Considerations

Total ankle arthroplasty (TAA) through the anterior approach is associated with wound-healing complication rates up to 28%.^20,30^ These can lead to infection and even amputation.^20^ Using CETE could support optimal wound closure by off-loading pressure.^20^

##### Clinical evidence

A series of 35 TAA closures augmented with DermaClose reported decreased wound-related complications compared with standard skin closure.^20^ The authors reported faster healing, less swelling, and improved final wound appearance with CETE versus without.

##### Consensus recommendations

In TAA, the CETE device can be applied after primary closure to off-load pressure from the incision.^20^ The tension controller should be rotated until sufficient force is applied to take tension off the suture line; it should not be fully tightened.

The CETE device can be used alone or in conjunction with incisional VAC.

#### Diabetic foot wounds

##### Considerations

Diabetes mellitus affects bone and soft tissue healing, potentially resulting in wound complications and impaired healing.^31^ Patients with diabetes are at risk of developing diabetic foot ulcers, which may necessitate amputation,^32,33^ and have a high risk of postoperative flap failure.^34^ In large wounds, standard primary closure may not be an option, and second intention healing may take weeks or months.^19^

##### Clinical evidence

A report described two cases using a CETE device to treat chronic foot wounds in patients with diabetes mellitus.^19^ In the first, DermaClose was used to close a 3-year wound resulting from a hallux and second digital amputation in a 59-year-old man. The wound closed completely after ∼3 months; concomitant immunosuppressive medication may have contributed to long closure time. The second patient, a 42-year-old man, underwent partial metatarsal resection and digital amputation after an infection. Several wound care treatments were attempted, but the wound failed to close after several months, leaving a large granulating defect. Final wound closure was achieved a few days after application of DermaClose.

A case report using Proxiderm™ (Progressive Surgical Products, Westbury, NY) noted that CETE in diabetic patients could prevent major amputations.^33^ A patient with gangrene and amputation of the big toe developed necrotizing fasciitis; an above-the-knee amputation was recommended but declined. After CETE, combined with intensive wound care, the wound healed successfully and amputation was avoided.

As detailed in the Head and Neck, Including Scalp section, a prospective case series included seven cases using DermaClose for scalp wounds.^35^ Primary closure was achieved in five of seven patients. One patient with poorly controlled diabetes, who previously underwent external beam radiation, experienced partial skin loss and required bilateral advancement rotational flaps.

##### Consensus recommendations

Selecting patients with good vascularity at the wound site is crucial for optimal long-term results. Chronic wounds must be thoroughly debrided and wound edges excised before applying CETE to convert them into fresh acute wounds.

Off-load the wound site and prevent walking on it for several weeks after closure to ensure the wound remains closed.

### Plastic surgery

#### Closure of flap donor sites

##### Considerations

Certain large surface area skin-based flaps, such as anterolateral thigh (ALT), deep inferior epigastric, thoracoacromial, groin, and random pattern flaps, have low donor site morbidity and good soft tissue availability. However, donor site issues can include pain, prolonged wound healing, and difficulty achieving immediate closure.^22, 25^

##### Clinical evidence

**Figure 8.**
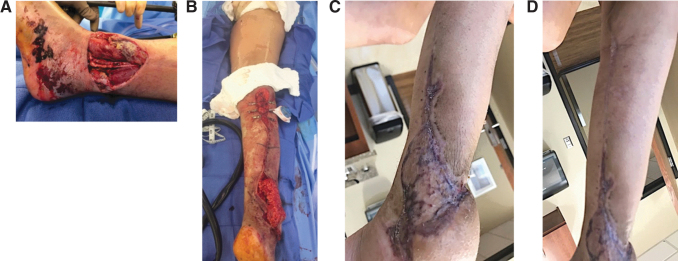
Donor site closure after reverse sural flap. **(A)** Traumatic wound. **(B)** Harvest of a reverse sural flap with application of DermaClose to close the donor site. **(C)** Healed flap site. **(D)** Healed donor site. Photographs courtesy of B.J.M.

CETE may be used for donor site closure after reverse sural flaps. One expert contributor has used this method in 11 patients. Average wound size was 149.9 cm^2^ (*n* = 10, range = 24–450 cm^2^). Six patients have completed donor wound treatment with an average healing time of 13 weeks (Table 3). No donor site complications have been noted at this point. A representative case from the series is given in Fig. 8. A teenager involved in a car accident sustained extensive injury to the ankle. A reverse sural artery rotational flap was performed, and DermaClose was used to close the donor site wound. A skin graft would typically be used, resulting in a large area with abnormal appearance. However, successful healing after CETE yielded a cosmetically favorable result.

**Table 3.** Reverse sural flap donor site wound and demographic information for patients who have completed donor wound treatment (n = 6)

Demographic			
Mean age	37.1 years		
Gender	33.3% male, 66.7% female		
*Wound Size*	*Donor Flap Type*	*Mean Time to Closure*	*Complications*
19–59 cm^2^	Reverse sural	18.0 weeks	None
60–99 cm^2^	Reverse sural	10.7 weeks	None
>100 cm^2^	Reverse sural	14.5 weeks	None

In a report of two patients with ALT free flaps, two complications were described.^25^ A 70-year-old man had limited knee extension after the use of DermaClose (timeframe not specified); the femoral nerve appeared intact but intramuscular electromyography showed minimal nerve function. Second, a 33-year-old woman with Crohn's disease had necrosis of the rectus femoris muscle 1 week after closure.

A study of the CETE device DynaClose (Canica Design, Inc., Almonte, Ontario) applied before radial forearm free-flap procedures found that primary closure of the donor site was associated with the lowest costs, followed by full- and split-thickness skin grafting.^36^ This is likely because of reduced healing time, lack of donor site, and low complication rates associated with CETE.

A retrospective review of DermaClose in ALT free-flap donor site wounds reported successful direct closure in 19 of 20 patients.^22^ Average flap width was 11.9 cm (range, 10–15 cm) and expansion time with CETE was 9.6 days (range, 4–18 days). Hospital stay length remained unchanged using CETE, and no adverse events associated with CETE were reported. Skin irritation and puncture marks caused by skin anchors were common but not problematic. Direct closure without skin graft was beneficial for amputees, as it allowed a more durable skin interface with the prosthesis.

##### Consensus recommendations

CETE can assist in closure of a flap donor site and possibly avoid the need for skin grafts or secondary flaps by supporting delayed primary closure of the donor wound.

#### Head and neck, including scalp

##### Considerations

The head, neck, and scalp can be challenging areas for surgery, because of cosmetic aspects (*i.e*., presence of hair-bearing skin) and physiology (nonelastic skin).^35^ Tissue expansion allows replacement with like-for-like tissue, potentially improving cosmetic appearance compared with other techniques.^35^

##### Clinical evidence

A retrospective, single-center study of six patients underwent definitive cranioplasty with preoperative CETE reported an average 16% increase in scalp surface area (range, 6.6–35.0%), resulting in all patients having adequate tissue for tension-free closure.^37^ The DermaClose device was well tolerated, and all patients were satisfied with the cosmetic outcome. No instances of delayed wound closure, infection, dermatitis, or cerebrospinal fluid leakage were reported. The device was applied an average of 238 days (standard deviation = ±60 days) after craniectomy, and the surgeon adjusted the device over the following 7–10 days.^37^

A prospective case series reported seven patients managed with CETE for scalp and forehead reconstruction after extirpation of malignant neoplasms.^35^ DermaClose was applied intraoperatively and remained for 6–14 days. Defect size was reduced by 50–99%, allowing 5 of 7 patients to achieve primary closure. One patient with poorly controlled diabetes required bilateral advancement rotation flaps, and another healed by secondary intention after device removal. Two patients experienced wound dehiscence after primary closure, 1–2 weeks after staple removal.

##### Consensus recommendations

One study reported placing the tension line circumferentially, with plastic tubes to protect the underlying skin.^37^ This may be useful in unique circumstances, but generally the authors consider the shoelace technique to be more effective.

Regarding the relative inelasticity of scalp tissue, galeal scoring is recommended to increase tissue mobility. Undermining of 2–3 cm from the tissue edges is standard.

In scalp wounds >5 cm long, using two CETE devices, each with six skin anchors, can be more effective and reduce closure time. In this situation, the skin anchors may be placed <1 cm apart.

### Patient age considerations

#### Considerations

Older patients may have chronic conditions such as cardiovascular disease, arthritis, thyroid disorders, and emphysema.^38^ Their skin may be especially fragile, because of deterioration of vasculature, collagen, and elastin.^38^

#### Clinical evidence

**Figure 9.**
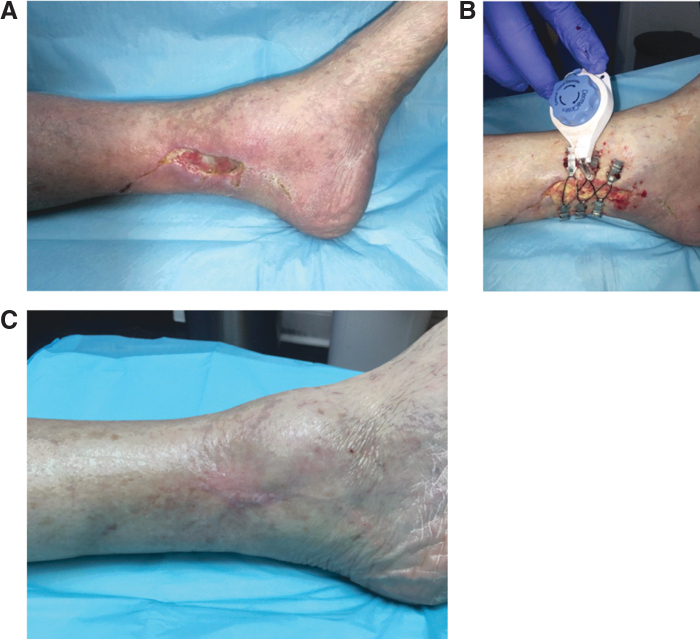
CETE in an elderly patient. **(A)** Wound at presentation. **(B)** Application of DermaClose^®^ device, which remained in place for 7 days. **(C)** Wound closed and healed at 3 months. Photographs courtesy of A.N.D.

In one representative case, a 77-year-old man presented with soft tissue loss (Fig. 9). A local flap was used to close the ankle wound, but it dehisced resulting in a chronic nonhealing wound. Primary closure was unsuccessful, and the wound was left open. DermaClose was applied, and the wound was reapproximated in 7 days.

A prospective case series reported the management of seven patients with a median age of 70 years (range, 57–87 years); see Head and Neck, Including Scalp section.^35^ No cases of skin breakdown around the skin anchors were observed.

#### Consensus recommendations

In older patients with questionable skin quality, the use of DuoDERM^®^ or similar type product under the skin anchors is encouraged to protect fragile skin. Place the skin anchors further from the wound edge than in younger patients, to avoid tearing.

### Orthopedic surgery

#### Technique modification

When using CETE over joints, the controller should be placed on one of the corners instead of by the middle anchor to avoid interfering with range of motion.

#### Trauma and amputations

##### Considerations

High-energy extremity trauma often leads to composite-type defects, which may have projectile penetration and a wide zone of injury. These wounds are frequently contaminated, which can lead to infection and necrosis despite significant debridement and numerous procedures.^18^ Frequently, primary closure is impossible, and adjunctive therapy is needed. Because of the often extensive nature of the injuries, it may be impossible to obtain sufficient tissue for a split-thickness skin graft.^39^

For amputations, limb length and viable joints should be preserved as much as possible.^40^ Patient compliance and adequacy of the implant rely on painless, durable soft-tissue coverage. This assists in successful ambulation in the case of lower limb amputations, and comfortable prosthetic fitting and wearing.^40^

##### Clinical evidence

**Figure 10.**
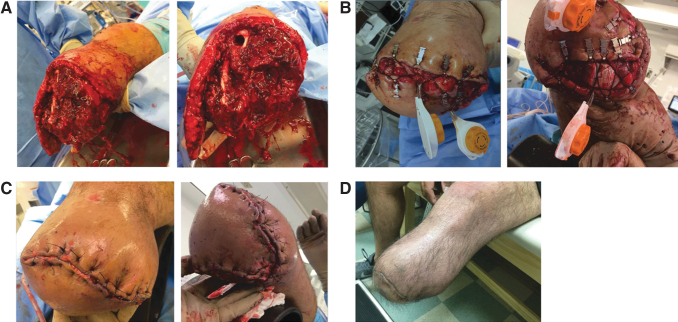
DermaClose in a complex closure. The patient sustained a traumatic below-the-knee amputation in an industrial accident. After operative debridement, an irregular pattern of soft tissue remained. CETE was utilized to facilitate closure using the long medial flap of skin to cover the amputation site. Simultaneous use of two CETE devices allowed complete closure of the amputation site with the patient's native tissue. **(A)** Wound after debridement. **(B)** Application of two CETE devices. **(C)** Closure of amputation site. **(D)** Wound completely healed. Photographs courtesy of B.J.M.

Figure 10 provides a case of a complex closure using DermaClose.

A case report briefly described a soldier with bilateral above-the-knee amputations and insufficient tissue for primary closure.^40^ DermaClose was used to enable soft tissue and dermal coverage, and the patient now ambulates with prosthetics.

See Fasciotomy section for a case report of a complicated fasciotomy after a gunshot wound to the left lower extremity.^28^

A retrospective review noted that direct closure without skin graft was beneficial for amputees, as it allowed a more durable skin interface with the prosthesis (see Closure of Flap Donor Sites section).^22^

A retrospective review of blast-related injuries treated with CETE reported successful delayed primary closure in 12 of 14 patients.^18^ The mean time to wound coverage was 4.4 days (range, 1–6 days). Two patients required split-thickness skin grafting to achieve definitive closure. No major complications were observed, but two patients experienced bullae or blister formation underneath the device, and three patients experienced maceration of the wound edge after device removal. Blisters were avoided in later patients by placing padding underneath the tension controller. Given its utility in treating soft tissue defects caused by traumatic injuries, triservice military treatment facilities and VA hospitals have continued to use DermaClose in hundreds of cases for complex wound closure.

##### Consensus recommendations

Complex wounds may require staged closing or serial CETE application. CETE may not be able to close the entire wound, but can be used as an adjunct to other wound management techniques, such as pedicle and rotational flaps and skin substitutes.

CETE may be particularly advantageous in cases of lower limb amputation, to preserve limb length without creating additional tissue defects.

#### Open fractures

##### Considerations

Gustilo–Anderson type IIIB open fractures are, by definition, associated with inadequate soft tissue coverage.^41^ Frequently, rotational and/or free flaps are used, but these require specialized care, prolonged hospital stay, and high cost.^41,42^ Management of these injuries hinges on durable soft tissue coverage, infection prevention, and bone healing without vascular compromise.

##### Clinical evidence

A report described successful wound healing in a 32-year-old man with an open Gustilo–Anderson type IIIB fracture of the tibia and fibula.^41^ DermaClose was applied after repeat irrigation, debridement, and partial wound closure. After 2 days, sufficient soft tissue was available for appropriate wound approximation.

A case report using Sure-Closure skin-stretching system successfully used stress–relaxation on forearm skin^42^ after open fracture of the left ulna. After open reduction and internal fixation, the soft tissue was swollen and prevented closure. However, after the stress–relaxation technique, the skin edges were brought together and the wound closed.

##### Consensus recommendations

Staged closing and serial application of the CETE device may be necessary to address wounds associated with open fractures. When other wound care methods are needed, CETE may be used as an adjunct treatment. As previously described, NPWT is useful for traumatic wounds, especially in conjunction with CETE.

## Summary

CETE can be used for wound management in multiple settings, and could be an addition to the traditional reconstructive ladder. Unlike other systems and devices, DermaClose applies constant tension and does not require tightening. There is clinical evidence supporting the use of this device in a number of wound types, and its use could avoid the need for a skin graft or other more invasive procedures.

As CETE is becoming more widely used, it is important to compile information on its efficacy and safety to ensure surgeons apply the optimal technique and select the most appropriate patients.

**Take-Home Messages**

- Surgeons of various specialties can use the continuous, controlled force external tissue expander to achieve closure of a range of wounds.
- There is clinical evidence to support CETE use in a number of wound types, including fasciotomy, trauma, amputation, and flap donor sites.
- CETE could represent a new rung on the reconstructive ladder, positioned below split thickness skin graft.
- CETE is easy to use, is not associated with severe complications, and may lead to cost savings.
- It is imperative that surgeons using CETE follow correct technique and select patients carefully.
- Prospective studies with sample sizes sufficient to establish statistical power and allow for direct comparisons are mostly absent in the literature assessing CETE. Further studies are needed to provide higher levels of evidence regarding its efficacy in treating difficult wounds.

## About the Authors

**Brendan J. MacKay, MD,** is a board-certified orthopedic surgeon currently serving as a hand and microvascular surgeon at Texas Tech University Health Sciences Center and the Director or Hand and Microvascular Surgery at University Medical Center. Residency: NYU Langone Orthopedic Hospital. Fellowship: Hand Center of San Antonio. **Anthony N. Dardano, DO,** is a board-certified in general and plastic surgery currently serving as a plastic surgeon at Florida Atlantic University College of Medicine. Residency: Sinai Hospital of Wayne State University. Fellowship: University of Texas Medical Branch. **Andrew M. Klapper, MD,** is a board-certified plastic surgeon currently serving as the plastic surgeon at Delray Medical Center and teaches at Florida Atlantic University. Residency: NYU Institute of Reconstructive Plastic Surgery. **Selene G. Parekh, MD,** is a board-certified orthopedic surgeon currently serving at Duke Orthopaedics. Residency: Hospital of the University of Pennsylvania. Fellowship: University of Pennsylvania. **Mohsin Q. Soliman, MD,** is a board-certified general surgeon specializing in advanced laparoscopic procedures and abdominal wall reconstruction. Residency: University of Nebraska Medical Center. **Ian L. Valerio, MD,** is a board-certified plastic surgeon currently serving at the Ohio State University College of Medicine. He is also Commander in the Medical Corps of the US Navy Active Reserve. Residency: University of Pittsburgh Medical Center.

## Abbreviations and Acronyms

ALT: anterolateral thigh
cAMP: cyclic adenosine monophosphate
CETE: continuous external tissue expansion
ECM: extracellular matrix
EGF: epidermal growth factor
NPWT: negative-pressure wound therapy
TAA: total ankle arthroplasty
VAC: vacuum-assisted closure
